# 415 Designing nanoparticle therapies for age-related macular degeneration – ERRATUM

**DOI:** 10.1017/cts.2026.10776

**Published:** 2026-06-30

**Authors:** Suleyman Bozal, Johnny Sheng Yue, Marcello DiStasio, Allison Rothrock, Xianzhi Zhang, Hande Eda Sutova, Sagar Bhatta, Emily Deschenes, Gyoung Ju, W. Mark Saltzman, Brian P. Hafler

**Affiliations:** 1 Yale School of Medicine, Yale University; 2 Yale University

When this was originally published in the Journal of Clinical and Translational Science it omitted to include the author Johnny Sheng Yue. This has been updated in the original article.

The publisher apologises for this error.
